# Supplementary material for: Impact of prenatal phthalate exposure on newborn metabolome and infant neurodevelopment
Source: Nat Commun. 2025 Apr 2;16:2539. doi: 10.1038/s41467-025-57273-z (PMC11965525; doi:10.1038/s41467-025-57273-z)
Supplement: Supplementary file 1 — Supplementary Information [file 41467_2025_57273_MOESM1_ESM.pdf]

Impact of Prenatal Phthalate Exposure on Newborn Metabolome and Infant Neurodevelopment

Susan S. Hoffman[1], Ziyin Tang[2], Anne Dunlop[3], Patricia A. Brennan[4], Thompson Huynh[2], Stephanie M. Eick[2], Dana B. Barr[2], Blake Rushing[5,6], Susan L. McRitchie[6], Susan Sumner[5,6], Kaitlin R. Taibl[2], Youran Tan[2], Parinya Panuwet[2], Grace E. Lee[2], Jasmin Eatman[2], Elizabeth J. Corwin[7], P. Barry Ryan[2], Dean P. Jones[8], Donghai Liang*[2,1]

[1]Department of Epidemiology, Emory University, Atlanta, GA, USA
[2]Gangarosa Department of Environmental Health, Emory University, Atlanta, GA, USA
[3]Department of Gynecology and Obstetrics, School of Medicine, Emory University, Atlanta, GA, USA
[4]Department of Psychology, Emory University, Atlanta, GA, USA
[5]Department of Nutrition, University of North Carolina at Chapel Hill, Chapel Hill, NC, USA
[6]Nutrition Research Institute, University of North Carolina at Chapel Hill, Kannapolis, NC, USA
[7]School of Nursing, Columbia University, New York, NY, USA
[8]School of Medicine, Emory University, Atlanta, GA, USA

**Corresponding author:*

Donghai Liang
Gangarosa Department of Environmental Health; Department of Epidemiology
Emory University Rollins School of Public Health
1518 Clifton Rd, Rm 2037
Atlanta, GA 30322, USA
donghai.liang@emory.edu

Supplementary material

Table S1. Metabolites of the selected diesters of phthalates[8] and their primary uses[94]

Phthalate Diester	Metabolite	Where it can be found
Diethyl phthalate (DEP)	Monoethyl phthalate (MEP)	Toothbrushes; automobile parts; tools; toys; food; packaging; cosmetics; insecticides; aspirin
Dibutyl phthalate (DBP)	Mono-n-butyl phthalate (MBP)	Nail polish; plasticizer; adhesive and printing ink additive
Di-iso-butyl phthalate (DiBP)	Monoisobutyl phthalate (MiBP)	Plasticizer; adhesive
Benzylbutyl phthalate (BBzP)	Monobenzyl phthalate (MBzP)	Plasticizer; sealants; paints
di-2-ethylhexyl phthalate (DEHP)	Mono-2-ethylhexyl phthalate (MEHP)	Plasticizer
Di-2-ethylhexyl phthalate (DEHP)	Mono-2-ethyl-5-oxohexyl phthalate (MEOHP)*	Plasticizer
Di-2-ethylhexyl phthalate (DEHP)	Mono-2-ethyl-5-hydroxyhexyl phthalate (MEHHP)*	Plasticizer
Di-2-ethylhexyl phthalate (DEHP)	Mono-2-ethly-5-carboxypentyl phthalate (MECPP)*	Plasticizer

*Secondary metabolite

Table S2. Characteristics of the Atlanta African American Maternal-Child Cohort, 2016-2018, compared to the sample population. The cohort sample consisted of 547 participants. The main sample was used for the metabolomic-wide association study (MWAS) and analyzed independently at two different time points (visit 1 n = 216; visit 2 n = 145). The subset was used for the meet-in-the-middle analysis, analyzed separately at the two study visits (visit 1 subset n = 81; visit 2 subset n = 71). Atlanta African American Maternal-Child cohort, 2016-2018.

**Characteristic**	**Cohort** n = 547	**Visit 1 (8-14 weeks)** Main sample = 216	Subset = 81	**Visit 2 (24-30 weeks)** Main sample = 145	Subset = 71
	Median (IQR)	Median (IQR)		Median (IQR)	

Age at enrollment	24.0 (21.0, 28.0)	24 (21.0, 29.0)	24.0 (21.0, 29.0)	24 (21.0, 29.0)	24 (21.0, 29.0)
Pre-pregnancy BMI (kg/m^2)	27 (22, 34)	28 (23, 34)	27 (24, 34)	26 (22, 33)	26 (23, 34)
Gestational Age	V1: 11.29 (9.43, 12.86)<br>V2: 26.00 (24.57, 28.14)	11.29 (9.29, 13.00)	11.57 (9.29, 13.29)	25.43 (24.57, 27.57)	25.43 (24.36, 27.36)
Creatinine (mg/dL)	#	178 (122, 228)	180 (128, 229)	139 (100, 202)	143 (100, 205)
	n (%)	n (%)		n (%)	
Education					
< High school	87 (16)	34 (16)	11 (14)	20 (14)	8 (11)
High school	213 (39)	91 (42)	35 (43)	58 (40)	31 (44)
Some college or more	247 (45)	91 (42)	35 (43)	67 (46)	32 (45)
Parity					
No prior birth	254 (46)	92 (43)	33 (41)	61 (42)	30 (42)
Prior birth	293 (54)	124 (57)	48 (59)	84 (58)	41 (58)
Alcohol use					
No	483 (88)	194 (90)	72 (89)	130 (90)	63 (89)
Yes	64 (12)	22 (10)	9 (11)	15 (10)	8 (11)
Tobacco use					
No	452 (83)	177 (82)	69 (85)	123 (85)	62 (89)
Yes	95 (17)	39 (18)	12 (15)	22 (15)	9 (13)
Marijuana use					
No	364 (67)	128 (59)	51 (63)	89 (61)	45 (63)
Yes	183 (33)	88 (41)	30 (37)	56 (39)	26 (37)
Sex of baby					
Male	257 (47)	102 (47)	43 (53)	68 (47)	35 (49)

Female	272 (50)	114 (53)	38 (47)	77 (53)	36 (51)
Unknown	18 (3)	0 (0)	0 (0)	0 (0)	0 (0)

BMI = body mass index (calculated as weight in kilograms divided by height in meters squared)
# = Data not available at the cohort level

Table S3. Exposure information in the subset selected for the meet-in-the-middle analysis (visit 1 n = 81; visit 2 n = 71). All phthalate metabolites were found in 100% of samples. Atlanta African American Maternal-Child cohort, 2016-2018.

**Metabolite (ng/ml)**	**Visit 1 (8-14 weeks) n = 81**		**Visit 2 (24-30 weeks) n = 71**	
	Median (IQR)	GM	Median (IQR)	GM
Monoethyl phthalate (MEP)	110 (39, 284)	114	86 (39, 211)	95
Mono-n-butyl phthalate (MBP)	11 (6, 23)	9.9	10 (4, 26)	9.1
Monoisobutyl phthalate (MiBP)	9 (3, 20)	9.1	8 (3, 17)	8.3
Monobenzyl phthalate (MBzP)	6 (3, 14)	6.3	5 (2, 13)	5.9
Mono-2-ethylhexyl phthalate (MEHP)	2 (0.7, 5)	1.7	2 (0.8, 4)	1.8
Mono-2-ethyl-5-oxohexyl phthalate (MEOHP)*	3 (2, 7)	3.4	3 (2, 7)	3.0
Mono-2-ethyl-5-hydroxyhexyl phthalate (MEHHP)*	6 (3, 11)	5.6	5 (2, 9)	4.0
Mono-2-ethly-5-carboxypentyl phthalate (MECPP)*	7 (4, 15)	8.6	7 (4, 11)	7.1
Sum of Di-2-ethylhexyl phthalate metabolites (ΣDEHP)	0.07 (0.03, 0.12)	0.07	0.05 (0.03, 0.1)	0.06

*Secondary metabolite; GM = geometric mean

Table S4. The number of metabolic features significantly associated ($p < 0.05$ or FDR corrected $q < 0.2$) with phthalate exposure levels in the full sample (visit 1 n = 216; visit 2 n = 145) using generalized linear models. These features were used for the pathway analysis and metabolite annotation. Source data is available in the Source Data files. Atlanta African American Maternal-Child cohort, 2016-2018.

**Exposure of interest**	**Significance threshold**	
	p < 0.05	q < 0.2
**MEP**		
Visit 1	224	0
Visit 2	260	14
**MBP**		
Visit 1	687	265
Visit 2	354	4
**MiBP**		
Visit 1	282	1
Visit 2	634	0
**MBzP**		
Visit 1	349	5
Visit 2	304	2
**MEHP**		
Visit 1	501	49
Visit 2	349	0
**MEOHP**		
Visit 1	289	3
Visit 2	426	29
**MEHHP**		
Visit 1	292	0
Visit 2	304	0

**MECPP**			
	Visit 1	394	1
	Visit 2	488	33
**ΣDEHP**			
	**Visit 1**	365	1
	**Visit 2**	393	12

Note: MEP = Monoethyl phthalate; MBP = Mono-n-butyl phthalate; MiBP = Monoisobutyl phthalate; MBzP = Monobenzyl phthalate; MEHP = Mono-2-ethylhexyl phthalate; MEOHP = Mono-2-ethyl-5-oxohexyl phthalate; MEHHP = Mono-2-ethyl-5-hydroxyhexyl phthalate; MECPP = Mono-2-ethly-5-carboxypentyl phthalate; ΣDEHP = Sum of Di-2-ethylhexyl phthalate metabolites.

Table S5. The number of metabolic features significantly associated ($p < 0.05$) with both phthalate exposure levels and NICU Net Neurobehavioral Scale (NNNS) scores in the subset (visit 1 subset n = 81; visit 2 subset n = 71) using generalized linear models. These features were used for the meet-in-the-middle analysis chemical annotation. Source data is available in the Source Data file. Atlanta African American Maternal-Child cohort, 2016-2018.

**Exposure of interest**		**Outcome**	
		Attention	Arousal
**MEP**			
	Visit 1	24	9
	Visit 2	10	19
**MBP**			
	Visit 1	21	16
	Visit 2	47	20
**MiBP**			
	Visit 1	18	10
	Visit 2	34	14
**MBzP**			
	Visit 1	6	16
	Visit 2	18	11
**MEHP**			

	Visit 1	11	11
	Visit 2	18	11
**MEOHP**			
	Visit 1	2	10
	Visit 2	13	12
**MEHHP**			
	Visit 1	4	8
	Visit 2	14	16
**MECPP**			
	Visit 1	4	11
	Visit 2	10	15
**ΣDEHP**			
	**Visit 1**	4	10
	**Visit 2**	9	11

Note: MEP = Monoethyl phthalate; MBP = Mono-n-butyl phthalate; MiBP = Monoisobutyl phthalate; MBzP = Monobenzyl phthalate; MEHP = Mono-2-ethylhexyl phthalate; MEOHP = Mono-2-ethyl-5-oxohexyl phthalate; MEHHP = Mono-2-ethyl-5-hydroxyhexyl phthalate; MECPP = Mono-2-ethly-5-carboxypentyl phthalate; ΣDEHP = Sum of Di-2-ethylhexyl phthalate metabolites.

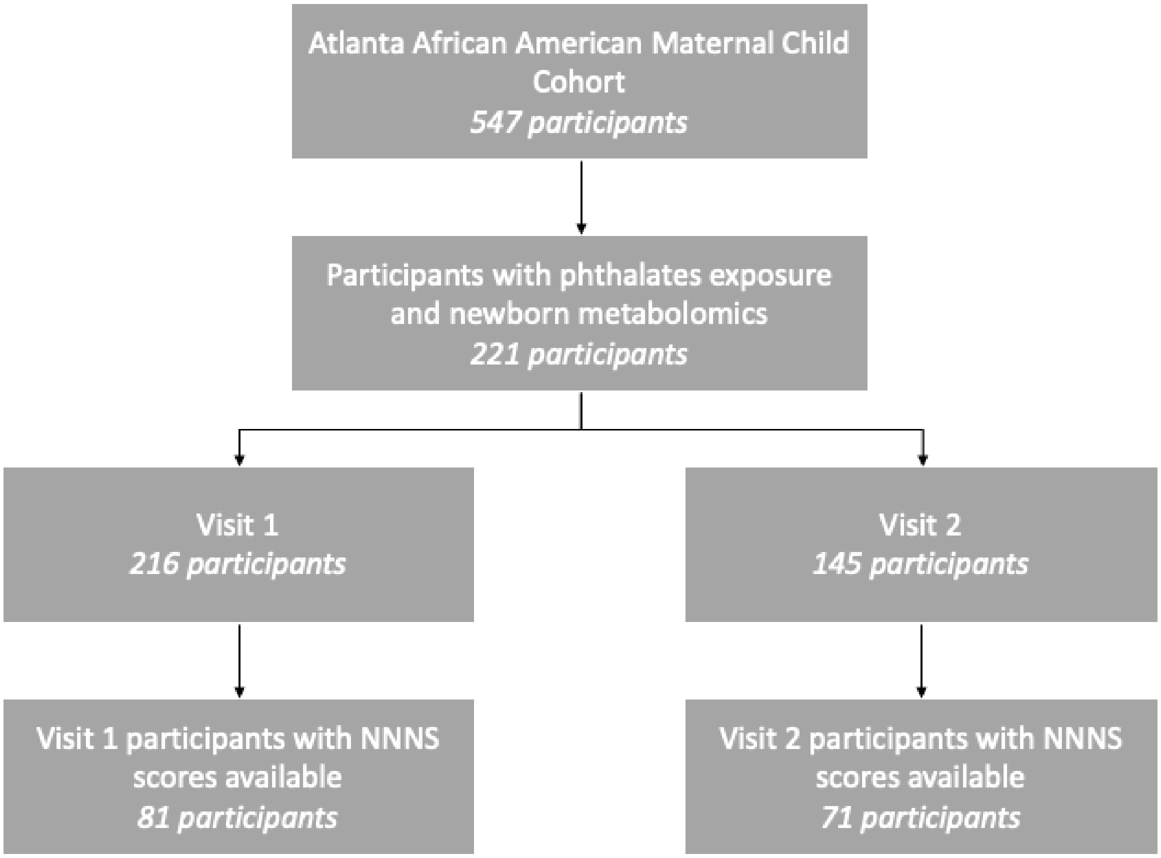

Figure S1. Population flowchart from the Atlanta African American Maternal-Child Cohort 2016-2018 (n=547) to the sample used in the metabolome-wide association study (MWAS) analysis (visit 1 n=216; visit 2 n=145) and the meet-in-the-middle (MITM) analysis (visit 1 n=81; visit 2 n=71)

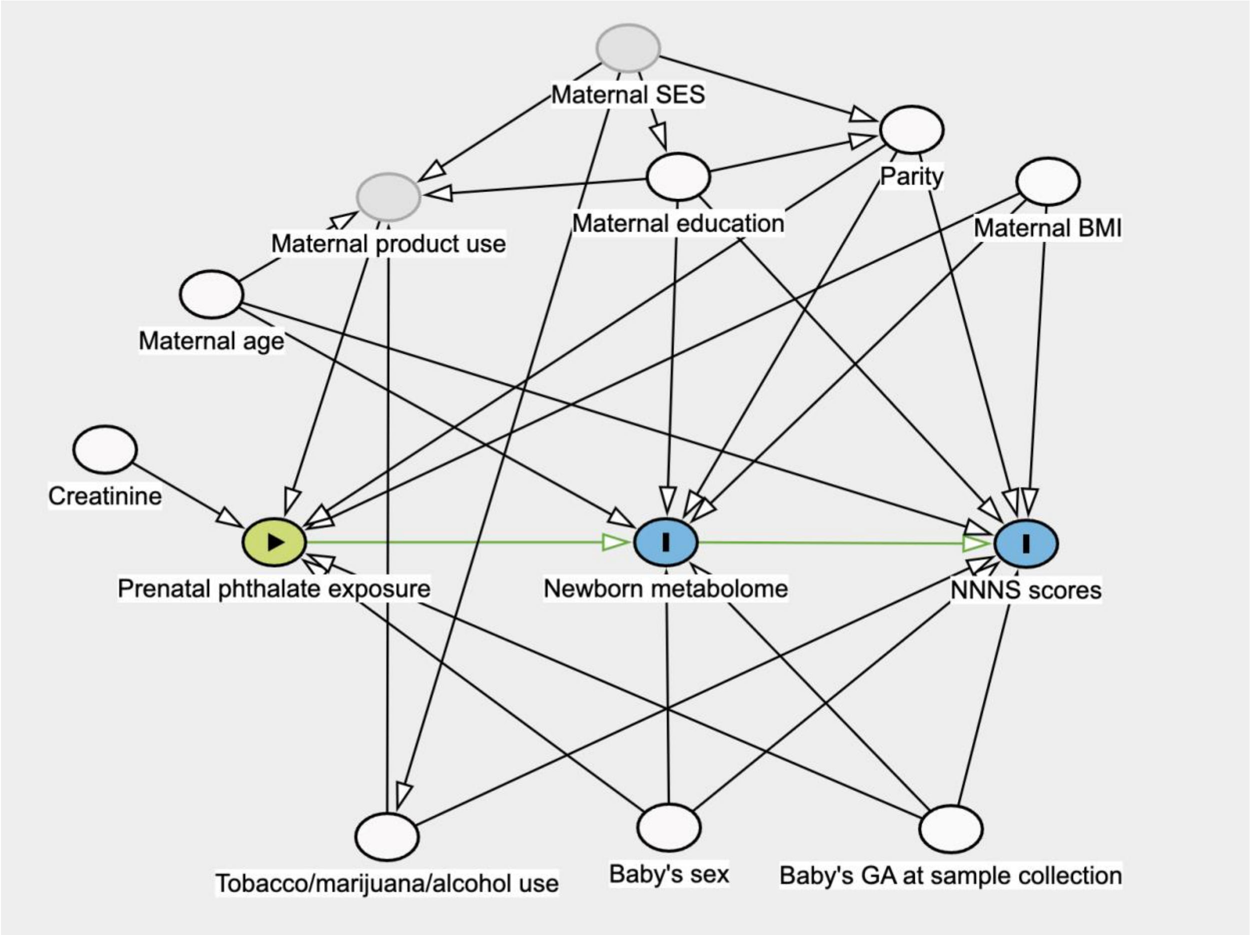

Figure S2. A directed acyclic graph (DAG) used for variable selection to control for confounding bias. Figure created using *dagitty* (https://www.dagitty.net/dags.html). Note: SES = socioeconomic status, GA = gestational age, BMI = body mass index, NNNS = NICU Net Neurobehavioral Scale

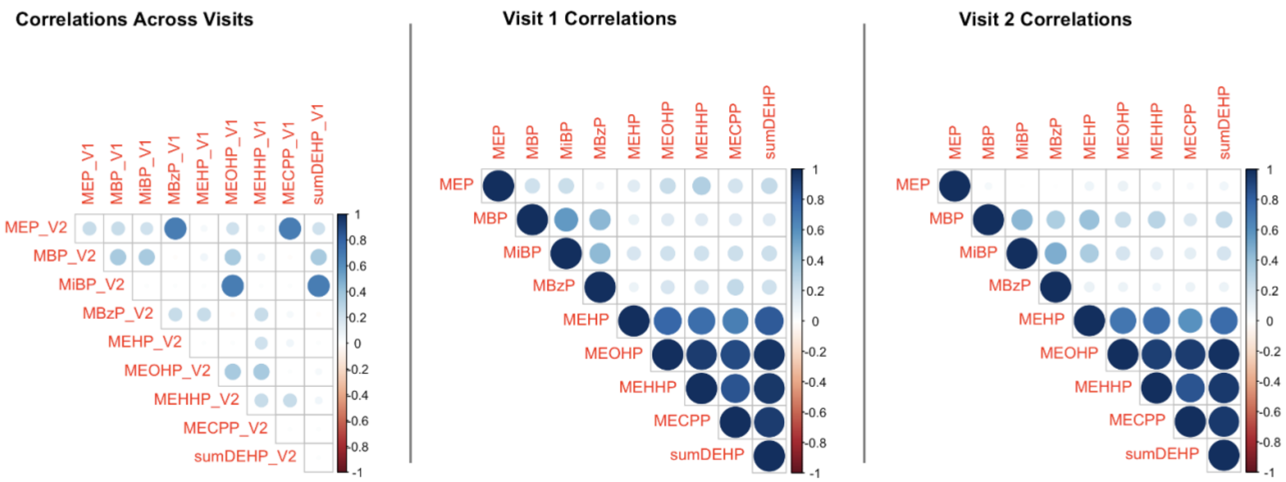

Figure S3. Correlations of phthalate metabolites across visits (n=140 matched participants) and within visits (visit 1 n = 216 and visit 2 n = 145)

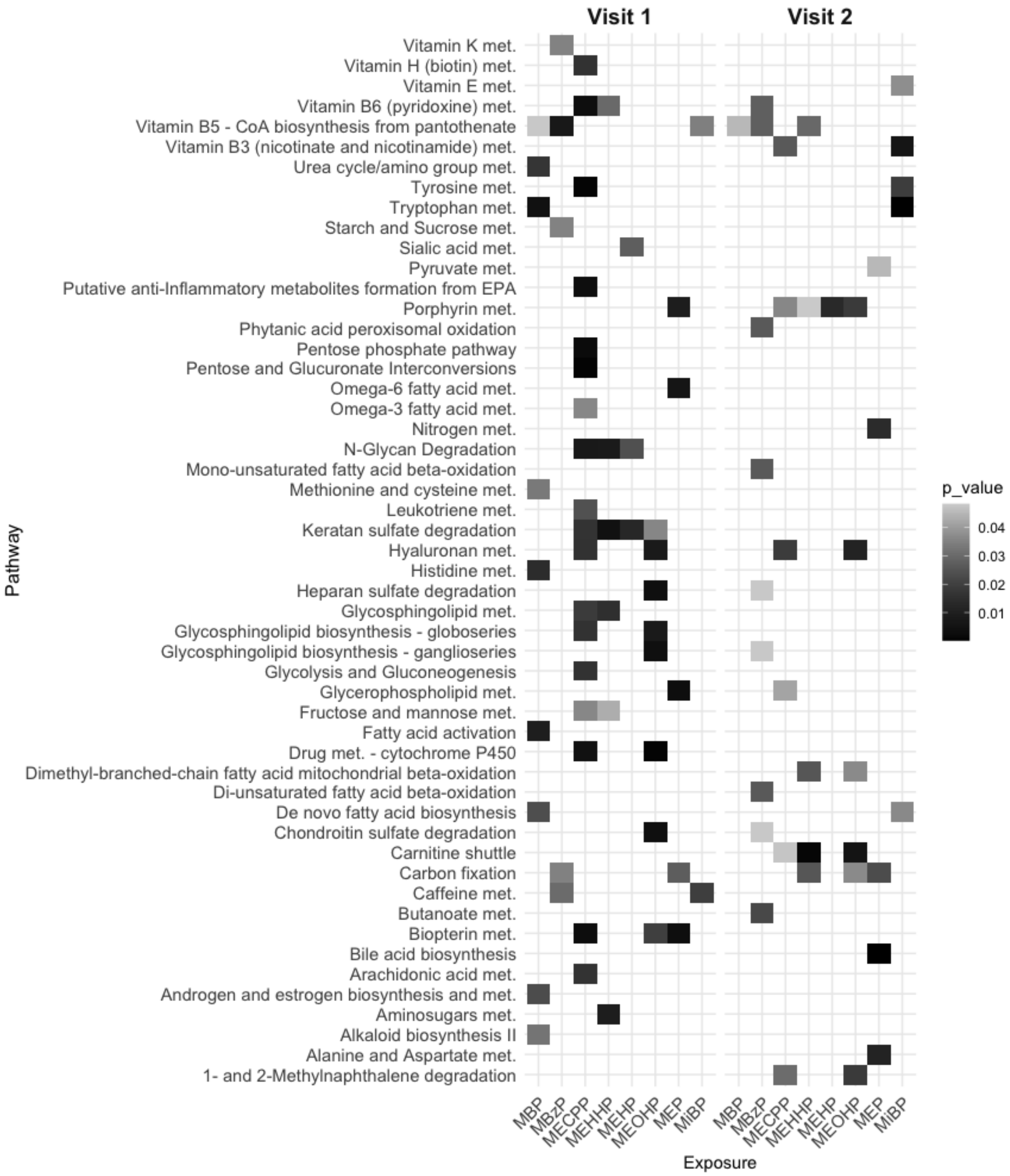

Figure S4. Biological pathways associated ($p < 0.05$) with at least one phthalate metabolite at visit 1 (n = 216) and visit 2 (n = 145) from the pathway analysis utilizing the bioinformatics tool, *mummichog*. Pathway names are listed along the Y-axis. Each phthalate metabolite is along the x-axis. The greyscale relates to the p-value significance, with a darker color denoting a lower p-value. Note: met. = metabolism; MEP = Monoethyl phthalate; MBP = Mono-n-butyl phthalate; MiBP = Monoisobutyl phthalate; MBzP = Monobenzyl phthalate; MEHP = Mono-2-ethylhexyl phthalate; MEOHP = Mono-2-ethyl-5-oxohexyl phthalate; MEHHP = Mono-2-ethyl-5-hydroxyhexyl phthalate; MECPP = Mono-2-ethly-5-carboxypentyl phthalate; ΣDEHP = Sum of Di-2-ethylhexyl phthalate metabolites. Atlanta African American Maternal-Child cohort, 2016-2018.
