# Supplementary material for: Impact of prenatal phthalate exposure on newborn metabolome and infant neurodevelopment
Source: Nat Commun. 2025 Apr 2;16:2539. doi: 10.1038/s41467-025-57273-z (PMC11965525; doi:10.1038/s41467-025-57273-z)
Supplement: Supplementary file 2 — Reporting Summary [file 41467_2025_57273_MOESM2_ESM.pdf]

nature portfolio

Corresponding author(s): Donghai Liang

Last updated by author(s): Jan 3, 2025

Reporting Summary

Nature Portfolio wishes to improve the reproducibility of the work that we publish. This form provides structure for consistency and transparency in reporting. For further information on Nature Portfolio policies, see our Editorial Policies and the Editorial Policy Checklist.

Statistics

For all statistical analyses, confirm that the following items are present in the figure legend, table legend, main text, or Methods section.

n/a	Confirmed	
☐	☒	The exact sample size (n) for each experimental group/condition, given as a discrete number and unit of measurement
☐	☒	A statement on whether measurements were taken from distinct samples or whether the same sample was measured repeatedly
☐	☒	The statistical test(s) used AND whether they are one- or two-sided<br>*Only common tests should be described solely by name; describe more complex techniques in the Methods section.*
☐	☒	A description of all covariates tested
☐	☒	A description of any assumptions or corrections, such as tests of normality and adjustment for multiple comparisons
☐	☒	A full description of the statistical parameters including central tendency (e.g. means) or other basic estimates (e.g. regression coefficient) AND variation (e.g. standard deviation) or associated estimates of uncertainty (e.g. confidence intervals)
☐	☒	For null hypothesis testing, the test statistic (e.g. F, t, r) with confidence intervals, effect sizes, degrees of freedom and P value noted<br>*Give P values as exact values whenever suitable.*
☒	☐	For Bayesian analysis, information on the choice of priors and Markov chain Monte Carlo settings
☒	☐	For hierarchical and complex designs, identification of the appropriate level for tests and full reporting of outcomes
☒	☐	Estimates of effect sizes (e.g. Cohen's d, Pearson's r), indicating how they were calculated

Our web collection on statistics for biologists contains articles on many of the points above.

Software and code

Policy information about availability of computer code

Data collection	All data collection were done on password-protected and encrypted tablet computers with REDCap software, which is a software that allows for the encrypted transfer of data to the Emory server over the internet in a HIPAA-compliant, encrypted manner.
Data analysis	All statistical analyses were conducted in R (Boston, MA, USA, Version 4.3.0). The UHPLC-HR-MS data were processed by Progenesis QI (version 2.1, Waters Corporation) for peak identification and alignment. Pathway enrichment analyses were performed on Python using the bioinformatics software Mummichog (Version 2.3), which predicts biological networks, pathways, and metabolites based on significant signals with tentative chemical identities.

For manuscripts utilizing custom algorithms or software that are central to the research but not yet described in published literature, software must be made available to editors and reviewers. We strongly encourage code deposition in a community repository (e.g. GitHub). See the Nature Portfolio guidelines for submitting code & software for further information.

Data

Policy information about availability of data

All manuscripts must include a data availability statement. This statement should provide the following information, where applicable:

- Accession codes, unique identifiers, or web links for publicly available datasets
- A description of any restrictions on data availability
- For clinical datasets or third party data, please ensure that the statement adheres to our policy

The raw and processed metabolomics data generated in this study have been deposited in the Metabolomics Workbench (https://

www.metabolomicsworkbench.org/ Study ID pending) by the UNC Human Health Exposure Analysis Resource (HHEAR) Laboratory. The clinical outcome and phthalate exposure data are available under restricted access to protect the privacy of the study participants, access can be obtained by emailing corresponding authors Drs. Liang and Dunlop with reasonable request. The demographic covariates data are protected and are not available due to data privacy laws. All the source data for figures and tables, coding materials, and data protocols are provided in the Supplementary Information/Source Data file.

Research involving human participants, their data, or biological material

Policy information about studies with human participants or human data. See also policy information about sex, gender (identity/presentation), and sexual orientation and race, ethnicity and racism.

Reporting on sex and gender	The study only recruited female pregnant people into the study and there was no information collected on the male partner. This is indicated by the use of "maternal" in both the title and abstract. As the newborns included both males and females, we were sure to use gender-inclusive language like "infant" and "newborn" throughout the manuscript and provided a breakdown of infant sex assigned at birth in Table 1. There were 114 (53%) newborns assigned female sex at birth. Neonatal sex was believed to impact the exposures and outcomes, and thus adjusted in the models in all analyses.<br><br>As we believe sex behaves as a confounder in this project and given the limited sample size underpowering any stratified analyses and complex analytical workflow, sex stratification was deemed to be inappropriate for this project. However, this point was emphasized in the discussion section as a future direction in larger-scale studies to determine what kind of an influence infant sex might have on this relationship.
Reporting on race, ethnicity, or other socially relevant groupings	Participants were eligible for the study if they self-reported African American or Black race, were US-born, 18-40 years old, pregnant with a singleton fetus between 8-14 weeks gestation, and did not have any diagnosis of chronic medical conditions or chronic use of prescription medications.<br><br>Covariates were selected through a thorough review of the literature and a directed acyclic graph (DAG) to identify covariates that could potentially introduce confounding bias. Mother's age has been shown to predict exposure levels of other environmental contaminants and can have impacts on newborn health outcomes. Education and parity were used as a proxy for socioeconomic status (SES). Women with lower SES have higher reported urinary phthalate metabolites, and there are well-established relationships between SES and adverse birth outcomes. Substance use (alcohol use, tobacco use, and marijuana use) is linked to increased phthalate exposure and has long-documented impacts on newborn health. BMI can change the metabolic processing of phthalates and can impact the neonatal metabolism. Creatinine was adjusted for in order to control for any differences that could arise in urine concentration between each of the participants. Baby's sex and gestational age at the time of sample collection were controlled for to avoid capturing differences driven by either the newborn's biological sex or the timing of the measured exposure window.
Population characteristics	The characteristics of 216 African American pregnant people and newborns included in our study are summarized in Table 1. Characteristics of participants with data from across the different visits and between the full sample MWAS and the subset were similar. Participants were in their mid-20s (median: 24, IQR: 21.0-29.0), had an overweight BMI (median: 28, IQR: 23-34) and most had a high-school education or higher. For most of the participants (57%), the pregnancy captured in this study was not their first. Participants did report substance use during pregnancy, with ~10% reporting alcohol use, ~15% reporting tobacco use, and ~40% reporting marijuana use.
Recruitment	Participants from the Atlanta African American Maternal-Child Cohort (ATL AA hereafter) were included in the present analysis. This ongoing, prospective birth cohort enrolls pregnant African Americans between 6–17 weeks gestation at Emory Midtown Hospital and Grady Hospital, which serve socioeconomically diverse populations in Atlanta, Georgia, and extends dyad follow-up through age six. Additional information regarding the cohort profile and data collection is described in detail elsewhere. Participants were eligible for inclusion if they self-identified as African American or Black, and were born in the US, between 18–40 years old, pregnant with a singleton, fluent in English, and had no chronic medical conditions. Participant data are confidential and proprietary information to the ATL AA cohort. All participants were recruited to participate in this study from the prenatal care clinics of two metropolitan hospitals in Atlanta, GA, affiliated with Emory University Woodruff Health Sciences Center: Grady Memorial Hospital, a county-supported hospital that serves as a safety net for low-income patients; and Emory University Hospital Midtown, a private hospital that serves patients from a wide economic range. There is no obvious self-selection bias or bias beyond those related to the potential difference among participants who wished to consent to participate in the study compared to those who did not, which is common in all prospective cohort study.
Ethics oversight	All participants provided written, informed consent to participate in the study, which was approved by the Institutional Review Board at Emory University (approval reference number 68441).

Note that full information on the approval of the study protocol must also be provided in the manuscript.

Field-specific reporting

Please select the one below that is the best fit for your research. If you are not sure, read the appropriate sections before making your selection.

☐ Life sciences ☐ Behavioural & social sciences ☒ Ecological, evolutionary & environmental sciences

For a reference copy of the document with all sections, see nature.com/documents/nr-reporting-summary-flat.pdf

Ecological, evolutionary & environmental sciences study design

All studies must disclose on these points even when the disclosure is negative.

Study description	In a well-established prospective cohort of pregnant African American people and their infants, we first sought to investigate the relationship between prenatal urinary phthalate levels and perturbations in the newborn metabolome. Based on the prior work, we also hypothesized that urinary phthalate levels may interfere with infant neurodevelopment (evaluated by NNNS scores) through perturbations in the newborn metabolome. To test this hypothesis, we further explored the potential mechanisms behind prenatal exposure to phthalates and neurobehavioral outcomes by identifying potential intermediate biomarkers between phthalate exposure and NNNS scores within a subset of the same population using a meet-in-the-middle (MITM) approach.
Research sample	The present study was designed to investigate and elucidate the molecular connection between maternal exposures to phthalates, newborn metabolic perturbations, and infant neurodevelopment among African American pregnant people, as they disproportionately suffer from a range of environmental exposures and adverse birth outcomes, compared to U.S. women of other races/ethnicities. As such, all participants in this study were participants enrolled in the Atlanta African American Maternal-Child (ATL AA) Cohort who self-identify as African American or Black. This ongoing, prospective birth cohort enrolls pregnant African Americans between 6–17 weeks gestation at Emory Midtown Hospital and Grady Hospital, which serve socioeconomically diverse populations in Atlanta, Georgia, and extends dyad follow-up through age six. The ATL AA cohort is generally representative of the socioeconomically diverse African American populations in Atlanta, Georgia. A criterion for inclusion in this cohort, is that the mother self-identifies as African American (i.e., a US-born Black woman) and between 18- and 40-years of age, who had previously enrolled in our Prenatal Cohort study and who gave permission for their data and biosamples to be used for environmental chemical assessments and omics profiling.
Sampling strategy	The study participants included in the MWAS were restricted to participants who had urine and blood samples available for the determination of urinary phthalate levels and serum metabolomics. The analysis identifying potential intermediate biomarkers between phthalate exposure and NNNS scores was further subset to those mother-newborn pairs for whom infant neurodevelopment information (evaluated by NNNS scores) was available.<br><br>In the main MWAS, we evaluated a total of 216 participants with urinary phthalate data from prenatal visit 1 and 145 participants with urinary phthalate data from prenatal visit 2. In the subset used for the MITM analysis (which required newborn metabolome and NNNs data), there were 81 participants with phthalate exposure data from prenatal visit 1 and 71 with phthalate exposure data from prenatal visit 2.
Data collection	Sociodemographic and gestational age at birth outcomes were collected from medical record abstraction by medical personnel. All data collection were done by the ATL AA Cohort study team led by Dr. Anne Dunlop on password-protected and encrypted tablet computers with REDCap software, which is a software that allows for the encrypted transfer of data to the Emory server over the internet in a HIPAA-compliant, encrypted manner. The participants contributed blood samples in the clinical visits, which were collected by the same study team and stored at the -80 freezer. Newborn DBS samples are routinely collected at time of birth for medical screening and public health surveillance by the Georgia Department of Public Health then archived for future biomonitoring purposes. The NNNS was administered when infants were on average 2 to 4 weeks old (corrected for gestational age a birth) by trained and certified research specialists.
Timing and spatial scale	The live births occurred between 2016 and 2018. Maternal samples were collected between gestation weeks 8-14 and 24-30; while the dried blood spots were collected from the newborn within 48 hours of their births. All the study participants lived in the metropolitan area in the city of Atlanta, Georgia, USA.
Data exclusions	Those without available maternal phthalate levels and metabolomic data were not included in the study population as this data represented the exposure and outcome. This exclusion was pre-established.
Reproducibility	This is a prospective observational study without experiment.
Randomization	Not applicable as there is no group assigned for exposure/intervention. For the metabolomics analysis and the targeted exposure assessment on phthalates study samples were randomized before sample preparation and data acquisition.
Blinding	All biological samples were blinded to the technician who performed the targeted exposure assessment and untargeted metabolomics profiling.

Did the study involve field work? ☐ Yes ☒ No

Reporting for specific materials, systems and methods

We require information from authors about some types of materials, experimental systems and methods used in many studies. Here, indicate whether each material, system or method listed is relevant to your study. If you are not sure if a list item applies to your research, read the appropriate section before selecting a response.

Materials & experimental systems

n/a	Involved in the study
☒	☐ Antibodies
☒	☐ Eukaryotic cell lines
☒	☐ Palaeontology and archaeology
☒	☐ Animals and other organisms
☒	☐ Clinical data
☒	☐ Dual use research of concern
☒	☐ Plants

Methods

n/a	Involved in the study
☒	☐ ChIP-seq
☒	☐ Flow cytometry
☒	☐ MRI-based neuroimaging

Plants

Seed stocks	N/A
Novel plant genotypes	N/A
Authentication	N/A
